# Suction *Versus* Nonsuction Drainage After Uniportal Video-Assisted Thoracoscopic Surgery: A Propensity Score-Matched Study

**DOI:** 10.3389/fonc.2021.751396

**Published:** 2021-10-26

**Authors:** Jian Zhou, Chuan Li, Quan Zheng, Chenglin Guo, Mengyuan Lyu, Qiang Pu, Hu Liao, Lunxu Liu

**Affiliations:** ^1^Department of Thoracic Surgery, West China Hospital, Sichuan University, Chengdu, China; ^2^West China School of Medicine, Sichuan University, Chengdu, China; ^3^Department of Laboratory Medicine, West China Hospital, Sichuan University, Chengdu, China

**Keywords:** suction, drainage, uniportal, surgery, lung cancer

## Abstract

**Background:**

Uniportal video-assisted thoracoscopic surgery (UniVATS) was utilized with a rapid growth. The evidence is sparse, however, on whether to add external suction to water-seal drainage for chest drainage after UniVATS. This retrospective propensity score-matched study aimed to identify the necessity of adding external suction to chest drainage after UniVATS.

**Methods:**

Patients with lung cancer who underwent UniVATS were included from our prospectively maintained database. Patients were divided into two cohorts based on the addition of external suction to postoperative water-seal drainage or not. Propensity score-matched analysis was performed to identify the impact of suction on chest tube duration, incidence of persistent air leak, hospital stay, and hospitalization cost. Multivariable model with interaction terms was constructed to identify impact of covariables on effect of suction.

**Results:**

The two cohorts matched well on baseline characteristics (nonsuction: 173; suction: 96). Compared with nonsuction group, suction group showed longer median chest tube duration (3 *vs.* 2 days, *p* = 0.003), higher incidences of persistent air leak (9.4% *vs.* 1.2%, *p* = 0.003), persistent drainage (16.8% *vs.* 5.8%, *p* = 0.007), and reduced drainage volume within first 3 postoperative days (386.90 *vs.* 504.78 ml, *p* = 0.011). Resection extent was identified to mediate the relationship between suction and chest tube drainage.

**Conclusions:**

These findings discouraged adding external suction to water-seal drainage after UniVATS regarding longer chest tube duration and more persistent air leak. Patients undergoing lobectomy would benefit more from water-seal drainage without external suction compared with those doing sublobectomy.

## Introduction

Simple water seal (nonsuction drainage) or addition of suction to simple water seal (suction drainage) were routinely used for chest drainage after pulmonary surgery (1). Studies have tried to shed light on the differences between the two methods in postoperative outcomes, such as chest tube duration and incidence of persistent air leak (PAL) (2–4). We previously found that the addition of suction to simple water seal increased chest tube duration following pulmonary surgery (5). Nevertheless, the benefit of external suction remains questionable among different studies, regarding heterogeneities of participants, study protocols, and surgery types.

The thoracic surgery continuously moved towards minimal invasive era. Uniportal video-assisted thoracoscopic surgery (UniVATS) has been experiencing rapid growth as a less-invasive approach compared with multiportal VATS (6–8). UniVATS requires different geometric characteristics in equipment placement, operation view, as well as smaller incision, which might affect postoperative outcomes compared with standard three-port VATS. UniVATS was reported to result in shorter chest tube duration and less postoperative complications (2, 9, 10). Nevertheless, it still needed further insight into this newly emerging approach. External suction on chest tube drainage, as part of perioperative management, is lack of evidence on its effect on patients’ postoperative outcomes after uniportal VATS.

Here, in order to evaluate the necessity for additional suction on chest drainage after UniVATS, we retrospectively enrolled the patients who underwent UniVATS for lung cancer, and compared the efficiency of suction drainage and nonsuction drainage on chest tube duration. We also performed an updated meta-analysis to identify the effects of the additional suction to simple water seal on the postoperative outcomes.

## Material and Methods

### Patient Enrolment

We enrolled the patients who underwent UniVATS for lung cancer from our prospectively maintained database in West China Hospital, a national high-volume healthcare center, from January 2009 to August 2019. For exclusion are the following (1): lung cancer was not the primary indication for surgery (2); combined with other surgery at the same time (3); conversion to thoracotomy; and (4) history of cardiothoracic surgery. This study was approved by the Institutional Ethics Committee for Clinical Research of West China Hospital, Sichuan University [No. 2020 (1098)]. The exemption of informed consent has been applied.

### Standard Operating Procedures

All the patients enrolled from our prospectively maintained database received standard operating procedure. Generally, a 3–5-cm incision was made in the fourth or fifth intercostal space at the anterior axillary line. Lobectomy, segmentectomy, or wedge resection was performed as appropriate. The parenchyma was divided with a stapler. If necessary, systematic or lobe-specific lymph node dissection was conducted after completion of pulmonary resection. A water submersion test was used to detect presence of air leak, and sutures were applied in case of significant air leak. Fibrin sealant was covered on the cutting surface of pulmonary parenchyma if necessary. One 20F chest tube (Yangzhou Hanjiang Huafei Medical Device Factory, Co., Ltd, Hanzhou, China) was inserted through the working incision before closing chest wall. The chest tube was connected to the simple water-seal bottle (nonsuction drainage) or external suction (−10 cmH_2_O) was added to water seal (suction drainage) with traditional chest drainage system. Suction was experimentally added to the chest drainage by doctors. The external suction was connected initially after surgery lasting for 2 days and then switched to simple water seal until the chest tube removal (see **Supplementary Material 1**). The removal criteria for both the two groups included less than 300 ml drainage fluid/day, no bubbling observed lasting 12 h, and complete lung expansion in chest radiology. If chest tube could be removed early with a duration shorter than 2 days, suction would remain until the chest tube removal. We assessed air leak and recorded chest drainage volume every day. Chest radiology was performed routinely to examine lung expansion and chest drainage on the first postoperative day. Patient was discharged the next day after chest tube removal as if no accident existed.

### Postoperative Outcomes

The primary outcome was chest tube duration. The secondary outcomes included incidence of postoperative PAL, drainage volume within the first 3 postoperative days, total drainage volume, length of postoperative hospital stay, and hospitalization cost. PAL was defined as persistent postoperative air leak longer than 5 days. The incidence of other postoperative complications was also analyzed, including pulmonary infection and persistent drainage. Persistent drainage was defined as chest tube drainage lasting longer than 5 days due to excess pleural drainage.

### Sample Size Calculation

We estimated sample size by taking the chest tube duration as the primary outcome. The effect size was estimated based on a previous study (11). The mean [standard deviation (SD)] chest tube duration in suction group and nonsuction group were 3.8 (2.1) and 2.7 days (1.1), respectively. The effect size was calculated to be 0.65. We set the type I error as 5%, statistical power as 99%, and allocation ration of two groups as 2:1. The sample size was estimated as 168 (112:56) using G*Power (3.1.9, University of Dusseldorf, Germany).

### Propensity Score-Matched Analysis

We performed propensity score-matched (PSM) analysis to create a cohort of patients who received external suction with baseline characteristics similar to those who received only simple water seal. A logistic regression model was used to calculate propensity scores. Variables related to patient and disease were determined as confounders, including demographic characteristics (age, sex, body mass index (BMI)), smoking status, the American Society of Anaesthesiologists (ASA) grades, Eastern Cooperative Oncology Group (ECOG) performance status, and operation details (resection extent, surgery duration, pleural adhesion, intralobular fissure, fibrin sealant use). We identified propensity score-matched pairs using a 2:1 nearest greedy neighbor matching algorithm with caliper width of 0.2 without replacement (12). Absolute standardized mean difference ≤0.1 of the covariates indicated balance between the two cohorts (13). PSM was performed with the “matchit” package in R (4.0.3, R Development Core Team, Vienna, Austria).

### Statistical Analyses

Continuous variables with normal contribution were presented as mean [standard deviation (SD)] and compared with Student’s *t*-test, while those with nonnormal contribution were presented as median [interquartile range (IQR)] and compared with Wilcoxon test. Categorical variables were given as count (proportions) and compared by Fisher’s exact test. Risk factors for chest tube duration and incidence of PAL were explored using univariable and multivariable regression analyses on the unmatched cohort. Risk factors that were either significant in the univariable analysis or determined to be clinically important were put into the multivariable analysis. We put BMI and surgery duration as both continuous and categorical variables into the model. BMI was categorized into <18.5, 18.5–24.9, and ≥25 (14, 15). Surgery duration was categorized into short and long durations by median surgery duration of our whole cohort. Resection extent was categorized as lobectomy and sublobectomy (segmentectomy and wedge resection). To identify if resection extent, surgery duration (categorical), and fibrin sealant use would mediate relationship between suction and outcomes, three interaction terms between suction and those variables were included in the multivariable model. Multivariable analyses were conducted using generalized linear regression model by “glm” package in R (see **Supplementary Material 2**). We performed Poisson regression on chest tube duration and logistic regression for incidence of PAL. Overall risk (OR), β-coefficients, and 95% confidence interval (CI) were obtained. Goodness-of-fit tests for multivariable regression were performed with Chi-square test for Poisson regression and Hosmer-Lemeshow test for logistic regression.

We performed subgroup analyses on the unmatched cohort regarding resection extent, surgery duration, and use of fibrin sealant to identify potential difference in outcomes of patients in specific subgroups. All tests were two sided. *p* < 0.05 was considered statistically significant. False-discovery rate correction was used to adjust for multiple comparisons after multivariable analysis and subgroup analysis. Statistical analyses were performed using R (4.0.3, R Development Core Team, Vienna, Austria).

### Systematic Review on Prior Studies

We performed a meta-analysis of previous studies comparing suction *vs.* nonsuction groups. We included randomized clinical trials studying effects of suction and nonsuction groups on chest drainage published before September 15, 2021. We also included results of this study into meta-analysis. We focused on the outcome of chest tube duration. The effect size was assessed by weighted mean difference (WMD). A random-effects model was used to pool results. Meta-analysis was performed using “meta” R package.

## Results

### Patient and Clinicopathological Characteristics

**Figure 1:**
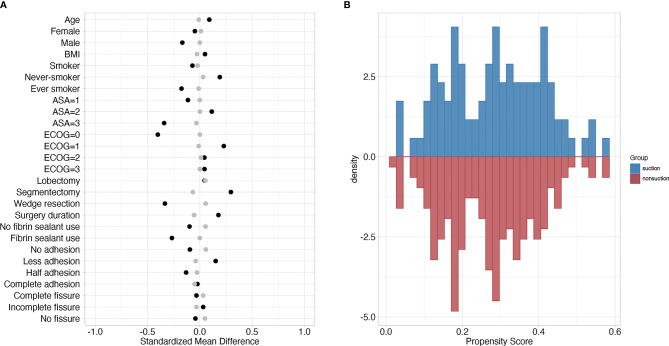
**(A)** Standardized mean differences of variables between suction and nonsuction groups. Black and grey dots represented standardized mean differences before and after matching, respectively. **(B)** Mirror histogram of propensity scores for suction group (above the x-axis) and nonsuction group (below the x-axis).

A total of 473 patients were finally included in the cohort. After PSM with a ratio of 2:1, the matched cohort included 269 patients (nonsuction drainage *vs.* suction drainage, 173 *vs.* 96) with well-balanced clinicopathological characteristics (**Figure 1**). **Table 1** showed the clinicopathological characteristics of patients before and after PSM.

**Table 1:** Baseline characteristics of the two groups before and after matching.

Characteristics	Before matching				After matching			
	Nonsuction (*n* = 371)	Suction (*n* = 102)	*p*-value	SMD	Nonsuction (*n* = 173)	Suction (*n* = 96)	*p*-value	SMD
Age (years)	55.99 (11.46)^a^	55.54 (10.85)	0.719	0.041	55.14 (10.30)	55.45 (11.08)	0.819	0.029
BMI (kg/m^2^)	22.94 (2.88)	22.85 (2.88)	0.787	0.023	22.90 (2.96)	22.70 (2.89)	0.585	0.07
Sex (female, %)	144 (38.8)	38 (37.3)	0.864	0.032	64 (37.0)	37 (38.5)	0.905	0.032
Smoking status (%)			0.412	0.155			0.981	0.025
Current	24 (6.5)	4 (3.9)			8 (4.6)	4 (4.2)		
Never	276 (74.4)	82 (80.4)			137 (79.2)	76 (79.2)		
Ever	71 (19.1)	16 (15.7)			28 (16.2)	16 (16.7)		
ECOG (%)			0.084	0.304			0.978	0.054
0	108 (29.1)	17 (16.7)			30 (17.3)	17 (17.7)		
1	259 (69.8)	83 (81.4)			140 (80.9)	77 (80.2)		
2	2 (0.5)	1 (1.0)			1 (0.6)	1 (1.0)		
3	2 (0.5)	1 (1.0)			2 (1.2)	1 (1.0)		
ASA (%)			0.102	0.253			0.944	0.043
1	21 (5.7)	2 (2.0)			4 (2.3)	2 (2.1)		
2	254 (68.5)	65 (63.7)			110 (63.6)	63 (65.6)		
3	96 (25.9)	35 (34.3)			59 (34.1)	31 (32.3)		
FEV1 (L)	2.60 (0.65)	2.60 (0.64)	0.946	0.049	2.62 (0.64)	2.59 (0.64)	0.738	0.043
FEV1/FVC (%)	80.18 (7.98)	80.82 (8.39)	0.484	0.09	80.28 (7.67)	80.49 (8.64)	0.841	0.025
Pleural adhesion (%)			0.376	0.213			0.969	0.064
None	205 (55.3)	48 (47.1)			91 (52.6)	48 (50.0)		
Minimal	128 (34.5)	44 (43.1)			65 (37.6)	39 (40.6)		
Half	27 (7.3)	6 (5.9)			11 (6.4)	6 (6.2)		
Diffuse	11 (3.0)	4 (3.9)			6 (3.5)	3 (3.1)		
Intralober fissure (%)			0.357	0.221			1.000	0.015
Fused	6 (1.6)	0 (0.0)			0 (0.0)	0 (0.0)		
Incomplete	188 (50.7)	49 (48.0)			86 (49.7)	47 (49.0)		
Complete	177 (47.7)	53 (52.0)			87 (50.3)	49 (51.0)		
Surgery duration (min)	112.77 (50.95)	99.69 (37.67)	0.016	0.287	103.98 (39.44)	101.47 (38.10)	0.613	0.065
Extent of resection			0.008	0.367			0.946	0.043
Lobectomy	170 (45.8)	49 (48.0)			80 (46.2)	45 (46.9)		
Segmentectomy	104 (28.0)	40 (39.2)			67 (38.7)	38 (39.6)		
Wedge resection	97 (26.1)	13 (12.7)			26 (15.0)	13 (13.5)		
Fibrin sealant use (%)	136 (36.7)	32 (31.4)	0.384	0.112	59 (34.1)	31 (32.3)	0.867	0.038

a Results are presented as mean (standard deviation) or counts (ratio). SMD, standard mean difference; BMI, body mass index; ECOG, Eastern Cooperative Oncology Group performance status; ASA, the American Society of Anaesthesiologists.

### Outcomes Related to Chest Drainage

Suction drainage showed increased median chest tube duration compared with nonsuction drainage [3 days (2–4) *vs.* 2 days (2–3), *p* = 0.003] (**Table 2**). However, the drainage volume within the first 3 postoperative days was lower in the suction group compared with the nonsuction group [386.90 ml (301.00) *vs.* 504.78 ml (387.90), *p* = 0.011]. The total volume of postoperative drainage was comparable between nonsuction drainage and suction drainage [605.92 ml (755.57) *vs.* 717.89 ml (849.13), *p* = 0.286].

**Table 2:** The postoperative outcomes in the unmatched and matched cohorts.

Outcomes	Unmatched			Matched		
	Nonsuction (371)	Suction (102)	*p*-value	Nonsuction (173)	Suction (96)	*p*-value
Chest tube duration (day)						
Median (IQR)	2 (2–4)	3 (2–4)	0.032	2 (2–3)^a^	3 (2–4)	0.003
Mean (SD)	2.99 (2.03)	3.51 (2.64)		2.80 (1.78)	3.63 (2.68)	
Drainage volume in first 3 postoperative days (ml)	511.09 (401.68)	388.21 (301.57)	0.004	504.78 (387.90)	386.90 (301.00)	0.011
Total drainage volume (ml)	786.38 (1169.10)	596.21 (739.05)	0.122	717.89 (849.13)	605.92 (755.57)	0.286
Postoperative length of hospital stays (days)	4 (3–6)	4 (3–6)	0.747	4 (3–5)	4 (3–6)	0.170
Total cost (¥)	48,773.54 (12,575.12)	48,413.08 (8,962.41)	0.787	48,974.10 (10,497.39)	48,736.85 (9,121.13)	0.853
Complications						
Pulmonary infection (%)	8 (2.2)	0 (0.0)	0.288	2 (1.2)	0 (0.0)	0.751
PAL (%)	10 (2.7)	9 (8.8)	0.012	2 (1.2)	9 (9.4)	0.003
Persistent drainage (%)	36 (9.8)	16 (15.8)	0.126	10 (5.8)	16 (16.8)	0.007

a Results are presented as mean (standard deviation), median (IQR), or counts (proportions). PAL, persistent air leak.

Both the incidence of PAL and persistent drainage was significantly lower in nonsuction group than that in the suction group [2 (1.2%) *vs.* 9 (9.4%), *p* = 0.003, 10 (5.8%) *vs.* 16 (16.8%), *p* = 0.007] (**Table 2**). No significant differences were identified in postoperative length of hospital stay, hospitalization cost, and the incidence of postoperative pulmonary infection.

### Regression Analysis

**Figure 2:**
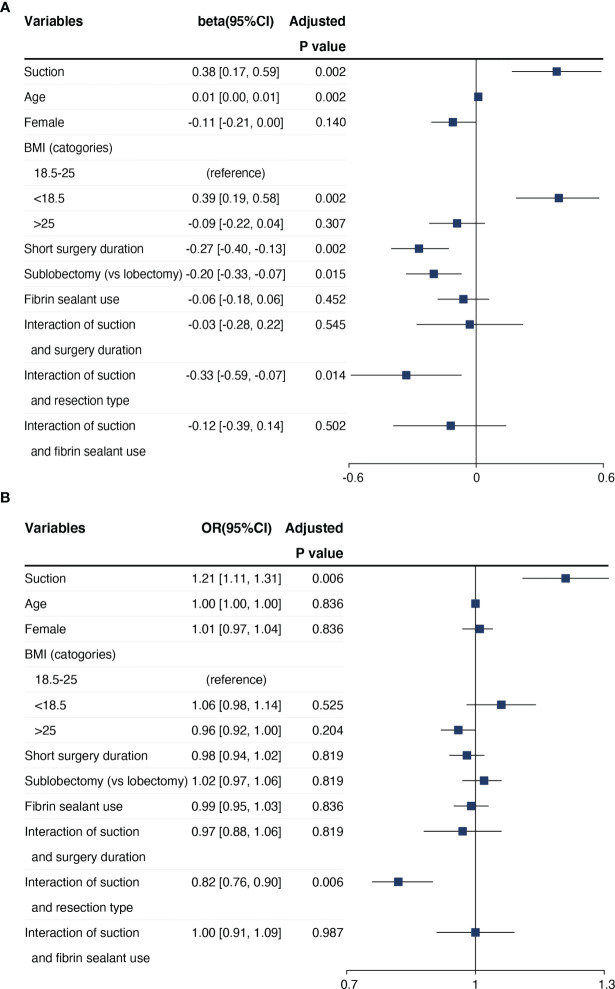
Results of multivariable regression analysis on the **(A)** chest tube duration and **(B)** incidence of PAL.

The goodness-of-fit test result for multivariable regression was *p* = 0.797 for chest tube duration and *p* = 0.699 for incidence of PAL. In the multivariable model for chest tube duration, resection extent was found to have a significant interaction with suction (**Figure 2**). After accounting for interactions, suction was also associated with increased chest tube duration (β = 0.38, 95% CI = 0.17 to 0.59, adjusted *p* = 0.002). Both sublobectomy (β = -0.20, 95% CI = -0.33 to -0.07, adjusted *p* = 0.015) and short surgery duration (β = -0.27, 95% CI = -0.40 to -0.13, adjusted *p* = 0.002) were associated with reduced chest tube duration. In the multivariable model for incidence of PAL, resection extent was also found to have a significant interaction with suction, while suction remained to be associated with increased incidence of PAL (OR = 1.21, 95% CI = 1.11 to 1.31, adjusted *p* = 0.006). However, suction was not an independent risk factor either in the multivariable analysis for drainage volume in the first 3 postoperative days and persistent drainage.

### Subgroup Analyses

**Figure 3:**
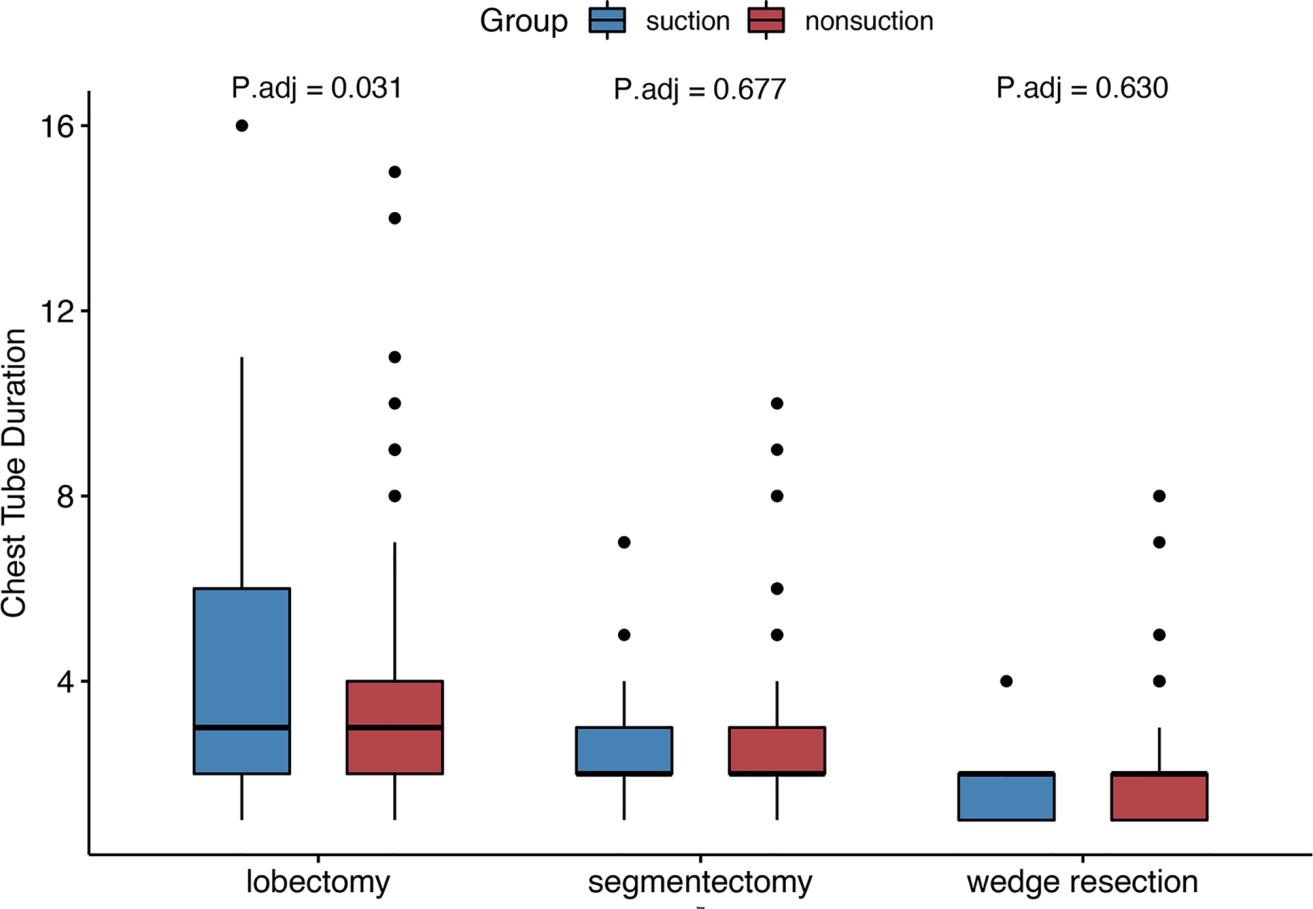
Subgroup analysis regarding extent of resection.

**Figure 4:**
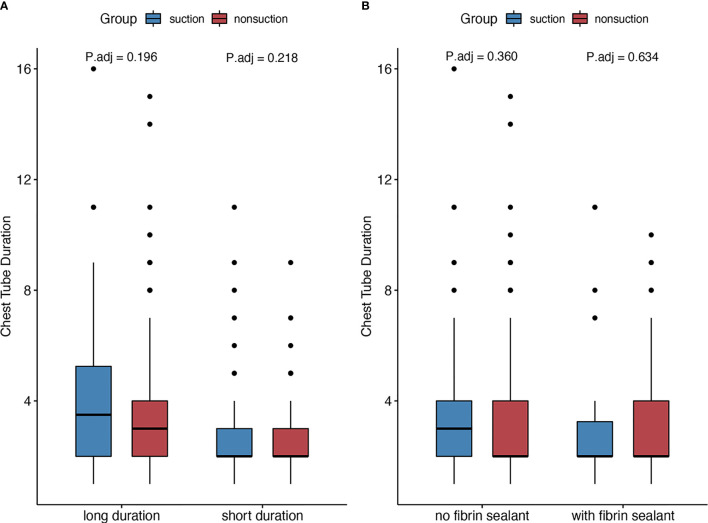
Subgroup analysis regarding **(A)** surgical duration and **(B)** intraoperative fibrin use.

In the patients who underwent lobectomy, patients with external suction showed increased chest tube duration (3 days (2–6) *vs.* 3 days (2–4), adjusted *p* = 0.031) and incidence of PAL [8 (20.0%) *vs.* 2 (2.8%), adjusted *p* = 0.008]. However, no significant differences were noticed in segmentectomy or wedge resection subgroups (**Figure 3**). The median surgery time was 105 min. Patients with shorter surgery time were considered shorter time group, and the others were considered longer time group. No significant increase was found in subgroups regarding surgery duration and fibrin sealant use (**Figure 4**).

### Meta-Analysis

**Figure 5:**
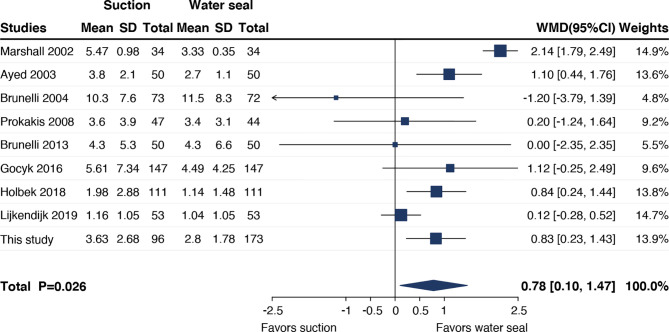
Meta-analysis on effect of addition of suction on chest tube duration.

A total of eight prior studies plus ours were included in the meta-analysis (3, 4, 11, 16–20). Results indicated that addition of suction significantly prolonged chest tube duration (WMD 0.78 days, 95% CI = 0.10 to 1.47, *p* = 0.026) (**Figure 5**).

## Discussion

We aimed to identify the efficiency of the addition of suction to simple water seal on postoperative chest drainage in patients who are undergoing UniVATS for lung cancer. We found significant differences between nonsuction drainage and suction drainage in terms of chest tube duration, drainage volume within the first 3 postoperative days, incidence of PAL, and persistent drainage. Multivariable analysis identified significant different effect of suction on outcomes between patients undergoing lobectomy and sublobectomy.

Compared with multiportal VATS, UniVATS was a less-invasive approach (6). Patients undergoing UniVATS resulted in less incidence of postoperative complications, shorter chest tube duration, and length of hospital stay compared with those doing multiportal VATS, indicating a faster recovery (2, 9, 10). Of note, the optimal chest drainage strategy still remains unanswered after UniVATS. To our knowledge, we are the first to focus on the patients undergoing UniVATS to identify the necessity of the additional suction to chest tube drainage.

Physiologically, applying external suction to simple water seal promoted the apposition between visceral and parietal pleurae, favoring sealing of air leak. On the other hand, negative pressure caused by suction might increase both the volume of air leak and the pleural liquid filtration. We found that suction drainage increased chest tube duration and was identified as an independent risk factor. This was consistent with a previous randomized trial (3). A meta-analysis involving four studies that also showed suction could result in increased chest tube duration (21). However, we found that suction group had less drainage volume within the first 3 postoperative days, which might be in conflict with increased chest tube duration. Yet, no significant difference existed in total drainage volume. A randomized trial revealed that suction increased postoperative drainage volume (3). Another trial showed higher suction pressure also resulted in increased chest drainage volume in patients undergoing three-port VATS (20). The addition of suction to simple water seal might hasten the evacuation of fluid, though at the same time the negative pressure might increase the fluid production. Under the circumstance of uniportal VATS, between the two conflict effects—fluid evacuation and production, the balance might be biased towards the former, leading to a decreased drainage volume in the suction drainage. However, chest tube duration in the suction group was still longer than that in the nonsuction group. It might be explained by our chest tube removal criteria. We withdrew chest tube based on two main criteria: (i) no air leak observed and (ii) no much fluid drained. The addition of suction to water seal was initially expected to accelerate chest tube removal. However, persistent air leak occasionally occurred, which might contribute to the delay of chest tube removal. Considering the ability of the pleura to absorb fluid, incidence of PAL might contribute more to prolonged chest tube duration than fluid output of chest drainage.

We found that the suction group showed a higher incidence of PAL compared with the nonsuction group, which is consistent with a previous study (3). Furthermore, suction drainage was found to be an independent risk factor for the increased incidence of PAL. This indicated that the addition of negative suction to simple water seal might aggravate air leak. The association of suction drainage with incidence of PAL is still controversial (3, 22), which might attribute to different surgery types, population selection, and PAL definition. Various definitions of PAL have been proposed as lasting postoperative air leak ranged from over 3 to 10 days (23), while the most widely acknowledged is over 5 days mainly according to the mean postoperative hospital stays nowadays (24). The definition threshold for persistent drainage followed the definition of PAL. Once PAL occurred, delayed chest tube removal and hospital discharge were inevitable (25). Also, we could choose endobronchial valves, sterile compressed sponge, or just supportive treatment without special intervention (26–28) to reduce the lasting of air leak.

For chest tube removal, we mainly judged the removal threshold by direct inspection of air bubbles in the water-seal chamber and recorded drainage volume based on traditional drainage system. Additionally, the digital chest drainage, a newly chest drainage system, could be used to guide chest tube removal (29). Digital drainage system was applied only in a few regions with high healthcare expenditure, while traditional drainage system was more widely used (3). We previously found that digital drainage system showed high effectiveness on postoperative recovery and might be worth of the promotion (30). Further studies on different drainage regimens with digital drainage system are well worthwhile.

Interestingly, according to the findings of multivariable analyses, patients undergoing lobectomy showed more effect from suction compared with those undergoing sublobectomy. This finding was further proved by the subgroup analysis, which showed significant differences in outcomes in lobectomy group but not in segmentectomy and wedge resection groups. It indicated that no addition of suction for chest drainage would benefit more on patients undergoing lobectomy compared with those doing sublobectomy. It might be because lobectomy resulted in a larger injury to lung parenchyma. The lung was more fragile and more easily to be interfered by the external suction. No similar results with the above existed in the variables of surgery duration and fibrin sealant use, both in multivariable analyses and subgroup analyses. BMI <18.5 was identified as an independent risk factor for prolonged chest tube duration. There was a hypothesis that lower BMI represented lower nutritional status and was detrimental to wound healing (15). As BMI was not identified as an independent predictor for incidence of PAL, it was included in the predictive models for PAL (31, 32). BMI <18.5 might also be taken into consideration when judging the application of suction drainage.

## Limitations

We have some limitations. Firstly, the retrospective design might bring selection bias and confounding bias, although we followed strict inclusion and exclusion criteria and applied PSM to balance potential confounding factors. Secondly, the limited number of patients enrolled in a single center might restrict the evidence power. The generalization of these results needs to be addressed carefully. Lastly, this study spanned 10 years, some changing techniques, such as the use of fibrin sealant (28) and discharge with a chest tube (33), were needed to be addressed when planning to use suction drainage. Herein, it warrants further investigations in prospective randomized controlled trials to explore the effect of addition of suction to simple water seal, setting of negative pressure in suction drainage, and stringent selection of benefited patients after UniVATS.

## Conclusion

In patients undergoing UniVATS for lung cancer, it might be unnecessary to add external suction to simple water-seal chest drainage after UniVATS for lung cancer. Patients undergoing lobectomy would benefit more from simple water seal on chest drainage without external suction.

## Data Availability Statement

The original contributions presented in the study are included in the article/**Supplementary Material**. Further inquiries can be directed to the corresponding authors.

## Ethics Statement

The studies involving human participants were reviewed and approved by the Institutional Ethics Committee on Biomedical Research, West China Hospital of Sichuan University [No. 2020 (198)]. Written informed consent for participation was not required for this study in accordance with the national legislation and the institutional requirements.

## Author Contributions

JZ, CL, and LL contributed to conception and design of the study. JZ and LL organized the database. CL, QZ, and ML performed the statistical analysis. JZ and CL wrote the first draft of the manuscript. CG, QP, and HL wrote sections of the manuscript. All authors contributed to manuscript revision and read and approved the submitted version.

## Funding

This work was supported by 1.3.5 Project for Disciplines of Excellence, West China Hospital, Sichuan University (ZYGD18021, ZYJC18009) (to LL), Post-Doctor Research Project, West China Hospital, Sichuan University (2020HXBH109) (to JZ), and 1.3.5 Project for Disciplines of Excellence-Clinical Research Incubation Project, West China Hospital, Sichuan University [2019HXFH060] (to QP).

## Conflict of Interest

The authors declare that the research was conducted in the absence of any commercial or financial relationships that could be construed as a potential conflict of interest.

## Publisher’s Note

All claims expressed in this article are solely those of the authors and do not necessarily represent those of their affiliated organizations, or those of the publisher, the editors and the reviewers. Any product that may be evaluated in this article, or claim that may be made by its manufacturer, is not guaranteed or endorsed by the publisher.

## Supplementary Material

Supplementary Material 1 Flowchart on drain management for suction and nonsuction groups.

Supplementary Material 2 R code for multivariable regression and production of forest plot of regression.

## References

[1] Brunelli A Beretta E Cassivi SD Cerfolio RJ Detterbeck F Kiefer T. Consensus Definitions to Promote an Evidence-Based Approach to Management of the Pleural Space. A Collaborative Proposal by ESTS, AATS, STS, and GTSC. Eur J Cardiothorac Surg (2011) 40(2):291–7. doi: 10.1016/j.ejcts.2011.05.020 21757129

[2] Gonzalez-Rivas D Paradela M Fernandez R Delgado M Fieira E Mendez L. Uniportal Video-Assisted Thoracoscopic Lobectomy: Two Years of Experience. Ann Thorac Surg (2013) 95(2):426–32. doi: 10.1016/j.athoracsur.2012.10.070 23219257

[3] Gocyk W Kużdżał J Włodarczyk J Grochowski Z Gil T Warmus J. Comparison of Suction Versus Nonsuction Drainage After Lung Resections: A Prospective Randomized Trial. Ann Thorac Surg (2016) 102(4):1119–24. doi: 10.1016/j.athoracsur.2016.04.066 27526655

[4] Lijkendijk M Licht PB Neckelmann K. The Influence of Suction on Chest Drain Duration After Lobectomy Using Electronic Chest Drainage. Ann Thorac Surg (2019) 107(6):1621–5. doi: 10.1016/j.athoracsur.2018.12.059 30742815

[5] Zhou J Chen N Hai Y Lyu M Wang Z Gao Y. External Suction Versus Simple Water-Seal on Chest Drainage Following Pulmonary Surgery: An Updated Meta-Analysis. Interact Cardiovasc Thorac Surg (2018) 28(1):29–36. doi: 10.1093/icvts/ivy216 30052997

[6] Bertolaccini L Batirel H Brunelli A Gonzalez-Rivas D Ismail M Ucar AM. Uniportal Video-Assisted Thoracic Surgery Lobectomy: A Consensus Report From the Uniportal VATS Interest Group (UVIG) of the European Society of Thoracic Surgeons (ESTS). Eur J Cardiothorac Surg (2019) 56(2):224–9. doi: 10.1093/ejcts/ezz133 31056711

[7] Rocco G Martin-Ucar A Passera E. Uniportal VATS Wedge Pulmonary Resections. Ann Thorac Surg (2004) 77(2):726–8. doi: 10.1016/s0003-4975(03)01219-0 14759479

[8] Hirai K Usuda J. Partial Lung Resection by Uniportal Video-Assisted Thoracoscopic Surgery: Technique and Pitfalls. Eur J Cardiothorac Surg (2020) 58(Suppl_1):i106–7. doi: 10.1093/ejcts/ezaa085 32243491

[9] Harris CG James RS Tian DH Yan TD Doyle MP Gonzalez-Rivas D. Systematic Review and Meta-Analysis of Uniportal Versus Multiportal Video-Assisted Thoracoscopic Lobectomy for Lung Cancer. Ann Cardiothorac Surg (2016) 5(2):76–84. doi: 10.21037/acs.2016.03.17 27134832 PMC4827401

[10] Wang B-Y Liu C-Y Hsu P-K Shih C-S Liu C-C. Single-Incision Versus Multiple-Incision Thoracoscopic Lobectomy and Segmentectomy: A Propensity-Matched Analysis. Ann Surg (2015) 261(4):793–9. doi: 10.1097/sla.0000000000000712 24836148

[11] Ayed AK. Suction Versus Water Seal After Thoracoscopy for Primary Spontaneous Pneumothorax: Prospective Randomized Study. Ann Thorac Surg (2003) 75(5):1593–6. doi: 10.1016/s0003-4975(02)04894-4 12735584

[12] Austin PC. Optimal Caliper Widths for Propensity-Score Matching When Estimating Differences in Means and Differences in Proportions in Observational Studies. Pharm Stat (2011) 10(2):150–61. doi: 10.1002/pst.433 PMC3120982 20925139

[13] Austin PC. Balance Diagnostics for Comparing the Distribution of Baseline Covariates Between Treatment Groups in Propensity-Score Matched Samples. Stat Med (2009) 28(25):3083–107. doi: 10.1002/sim.3697 PMC3472075 19757444

[14] Obesity WHOCo World Health O. Obesity: Preventing and Managing the Global Epidemic: Report of a WHO Consultation. Geneva: World Health Organization (2000). 11234459

[15] Thomas PA Berbis J Falcoz P-E Le Pimpec-Barthes F Bernard A Jougon J. National Perioperative Outcomes of Pulmonary Lobectomy for Cancer: The Influence of Nutritional Status†. Eur J Cardiothorac Surg (2013) 45(4):652–9. doi: 10.1093/ejcts/ezt452 24062351

[16] Marshall MB Deeb ME Bleier JI Kucharczuk JC Friedberg JS Kaiser LR. Suction *vs* Water Seal After Pulmonary Resection: A Randomized Prospective Study. Chest (2002) 121(3):831–5. doi: 10.1378/chest.121.3.831 11888968

[17] Brunelli A Monteverde M Borri A Salati M Marasco RD Al Refai M. Comparison of Water Seal and Suction After Pulmonary Lobectomy: A Prospective, Randomized Trial. Ann Thorac Surg (2004) 77(6):1932–7. doi: 10.1016/j.athoracsur.2003.12.022 15172239

[18] Prokakis C Koletsis EN Apostolakis E Panagopoulos N Kouki HS Sakellaropoulos GC. Routine Suction of Intercostal Drains Is Not Necessary After Lobectomy: A Prospective Randomized Trial. World J Surg (2008) 32(11):2336–42. doi: 10.1007/s00268-008-9741-3 18787890

[19] Brunelli A Salati M Pompili C Refai M Sabbatini A. Regulated Tailored Suction *vs* Regulated Seal: A Prospective Randomized Trial on Air Leak Duration. Eur J Cardiothorac Surg (2013) 43(5):899–904. doi: 10.1093/ejcts/ezs518 23024236

[20] Holbek BL Christensen M Hansen HJ Kehlet H Petersen RH. The Effects of Low Suction on Digital Drainage Devices After Lobectomy Using Video-Assisted Thoracoscopic Surgery: A Randomized Controlled Trial†. Eur J Cardiothorac Surg (2019) 55(4):673–81. doi: 10.1093/ejcts/ezy361 30445572

[21] Lang P Manickavasagar M Burdett C Treasure T Fiorentino F. Suction on Chest Drains Following Lung Resection: Evidence and Practice Are Not Aligned. Eur J Cardiothorac Surg (2016) 49(2):611–6. doi: 10.1093/ejcts/ezv133 25870218

[22] Leo F Duranti L Girelli L Furia S Billè A Garofalo G. Does External Pleural Suction Reduce Prolonged Air Leak After Lung Resection? Results From the AirINTrial After 500 Randomized Cases. Ann Thorac Surg (2013) 96(4):1234–9. doi: 10.1016/j.athoracsur.2013.04.079 23866802

[23] Attaar A Tam V Nason KS. Risk Factors for Prolonged Air Leak After Pulmonary Resection: A Systematic Review and Meta-Analysis. Ann Surg (2020) 271(5):834–44. doi: 10.1097/sla.0000000000003560 31577547

[24] Fernandez FG Falcoz PE Kozower BD Salati M Wright CD Brunelli A. The Society of Thoracic Surgeons and the European Society of Thoracic Surgeons General Thoracic Surgery Databases: Joint Standardization of Variable Definitions and Terminology. Ann Thorac Surg (2015) 99(1):368–76. doi: 10.1016/j.athoracsur.2014.05.104 25555970

[25] Khoury AL Kolarczyk LM Strassle PD Feltner C Hance LM Teeter EG. Thoracic Enhanced Recovery After Surgery: Single Academic Center Observations After Implementation. Ann Thorac Surg (2021) 111(3):1036–43. doi: 10.1016/j.athoracsur.2020.06.021 32805268

[26] Dugan KC Laxmanan B Murgu S Hogarth DK. Management of Persistent Air Leaks. Chest (2017) 152(2):417–23. doi: 10.1016/j.chest.2017.02.020 PMC6026238 28267436

[27] Hance JM Martin JT Mullett TW. Endobronchial Valves in the Treatment of Persistent Air Leaks. Ann Thorac Surg (2015) 100(5):1780–5; discussion 5-6. doi: 10.1016/j.athoracsur.2015.05.073 26294347

[28] Brunelli A Bölükbas S Falcoz PE Hansen H Jimenez MF Lardinois D. Exploring Consensus for the Optimal Sealant Use to Prevent Air Leak Following Lung Surgery: A Modified Delphi Survey From The European Society of Thoracic Surgeons. Eur J Cardiothorac Surg (2020) 59(6):1265–71. doi: 10.1093/ejcts/ezaa428 33337471

[29] Aldaghlawi F Kurman JS Lilly JA Hogarth DK Donington J Ferguson MK. A Systematic Review of Digital *vs* Analog Drainage for Air Leak After Surgical Resection or Spontaneous Pneumothorax. Chest (2020) 157(5):1346–53. doi: 10.1016/j.chest.2019.11.046 31958444

[30] Zhou J Lyu M Chen N Wang Z Hai Y Hao J. Digital Chest Drainage Is Better Than Traditional Chest Drainage Following Pulmonary Surgery: A Meta-Analysis. Eur J Cardiothorac Surg (2018) 54(4):635–43. doi: 10.1093/ejcts/ezy141 29659768

[31] Attaar A Winger DG Luketich JD Schuchert MJ Sarkaria IS Christie NA. A Clinical Prediction Model for Prolonged Air Leak After Pulmonary Resection. J Thorac Cardiovasc Surg (2017) 153(3):690–9.e2. doi: 10.1016/j.jtcvs.2016.10.003 27912898 PMC5651171

[32] Brunelli A Varela G Refai M Jimenez MF Pompili C Sabbatini A. A Scoring System to Predict the Risk of Prolonged Air Leak After Lobectomy. Ann Thorac Surg (2010) 90(1):204–9. doi: 10.1016/j.athoracsur.2010.02.054 20609776

[33] Bao F Dimitrovska NT Hu S Chu X Li W. Safety of Early Discharge With a Chest Tube After Pulmonary Segmentectomy. Eur J Cardiothorac Surg (2020) 58(3):613–8. doi: 10.1093/ejcts/ezaa097 32236542

