# Supplementary figures and images for: Suction Versus Nonsuction Drainage After Uniportal Video-Assisted Thoracoscopic Surgery: A Propensity Score-Matched Study
Source: Front Oncol. 2021 Oct 26;11:751396. doi: 10.3389/fonc.2021.751396 (PMC8577848; doi:10.3389/fonc.2021.751396)

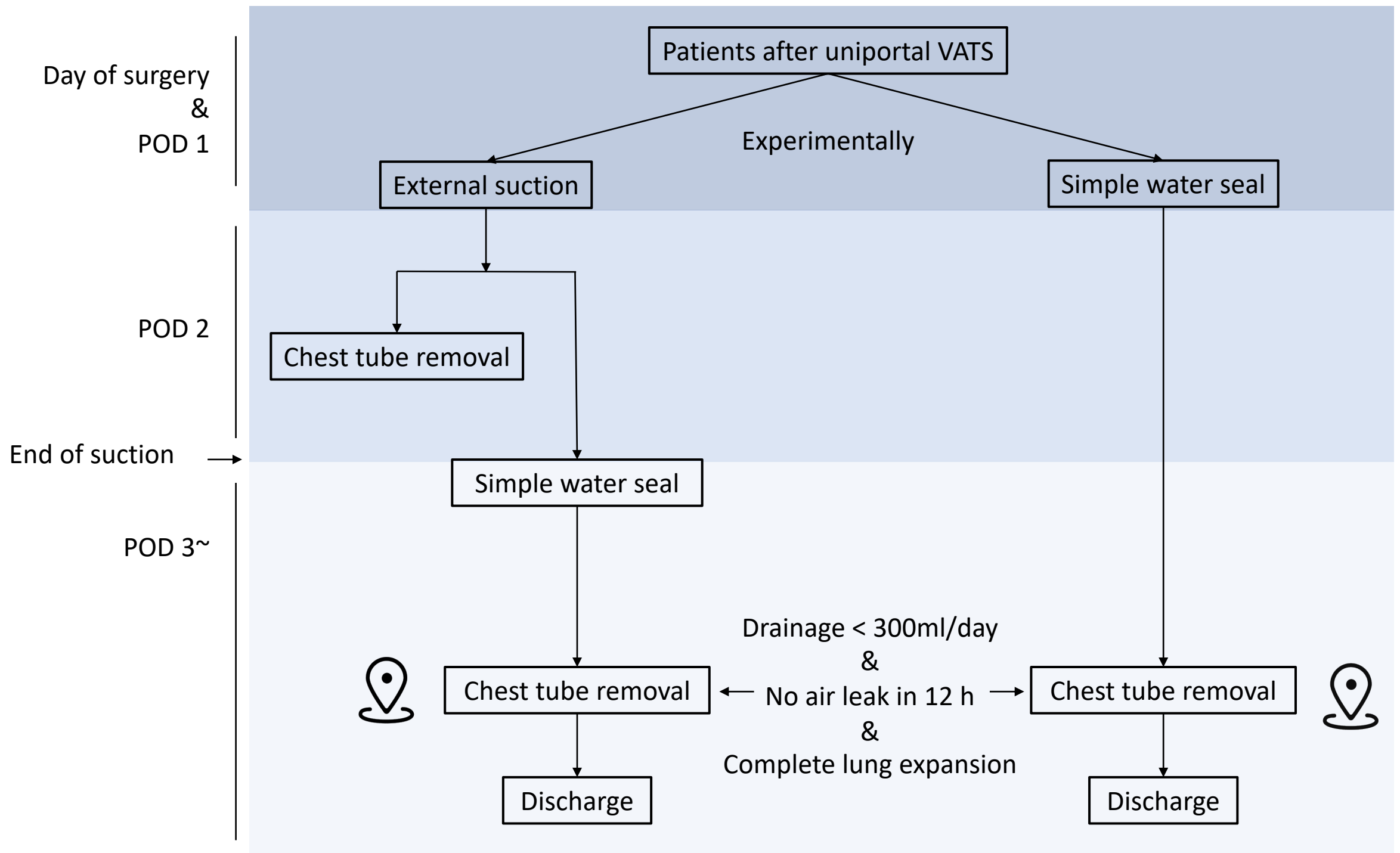

Supplement: Supplementary Material 1 — Flowchart on drain management for suction and nonsuction groups. [file DataSheet_1.pdf]
