# Supplementary material for: Suction Versus Nonsuction Drainage After Uniportal Video-Assisted Thoracoscopic Surgery: A Propensity Score-Matched Study
Source: Front Oncol. 2021 Oct 26;11:751396. doi: 10.3389/fonc.2021.751396 (PMC8577848; doi:10.3389/fonc.2021.751396)
Supplement: Supplementary Material 2 — R code for multivariable regression and production of forest plot of regression. [file DataSheet_2.docx]

options(stringsAsFactors = F)

library(glmnet)

library(survival)

library(tableone)

library(dplyr)

###..............................###

#1.regression

###..............................###

#1.1chestTubeDuration

mul_models <- glm(chestTubeDuration ~ age + sex + surgeryDuration*suction +

resectionType*suction + BMI + Naiwei*suction,family = gaussian(),

data = datt)

mulRes <- data.frame(ShowRegTable(mul_models,exp = F))

write.table(mulRes, file = './csvfile/mulResDurationAllwithInter.csv', sep = ',',

row.names = T,col.names = T)

#1.2PAL

datt$PD <- as.numeric(datt$PD)

mul_models2 <- glm(PD ~ age + sex + surgeryDuration*suction +

resectionType*suction + BMI + Naiwei*suction,family = logit(),

data = datt)

mulRes2 <- data.frame(ShowRegTable(mul_models2))

write.table(mulRes2, file = './csvfile/mulResPDallwithInter.csv', sep = ',',

row.names = T,col.names = T)

###..............................###

#2.forest_plot

###..............................###

list <- list("3" = gpar(lwd=2,

lineend="butt", columns=c(1:4), col="black"),

"4" = gpar(lwd=1,

lineend="butt", columns=c(1:3), col="#99999922"),

"5" = gpar(lwd=1,

lineend="butt", columns=c(1:3), col="#99999922"),

"6" = gpar(lwd=1,

lineend="butt", columns=c(1:3), col="#99999922"),

"7" = gpar(lwd=1,

lineend="butt", columns=c(1:3), col="#99999922"),

"8" = gpar(lwd=1,

lineend="butt", columns=c(1:3), col="#99999922"),

"9" = gpar(lwd=1,

lineend="butt", columns=c(1:3), col="#99999922"),

"10" = gpar(lwd=1,

lineend="butt", columns=c(1:3), col="#99999922"),

"11" = gpar(lwd=1,

lineend="butt", columns=c(1:3), col="#99999922"),

"12" = gpar(lwd=1,

lineend="butt", columns=c(1:3), col="#99999922"),

"13" = gpar(lwd=1,

lineend="butt", columns=c(1:3), col="#99999922"),

"15" = gpar(lwd=1,

lineend="butt", columns=c(1:3), col="#99999922"),

"17" = gpar(lwd=1,

lineend="butt", columns=c(1:3), col="#99999922"))

library(forestplot)

pl <- read.csv('./rdata/plot_data/pal_plot.csv', header = F)

forestplot(tt,

mean = pl$V4,

lower = pl$V5,

upper = pl$V6,

is.summary = c(T,T,F,F,F,F,F,F,F,F,F,F,F,F,F,F,F,F),

boxsize = 0.3,

lineheight = unit(8,'mm'),

colgap = unit(1,'mm'),

lwd.zero = 1,

lwd.ci = 1,

col = fpColors(box= '#1F3864',summary = '#1F3864', lines = 'black',

zero = 'black'),

lwd.xaxis= 1,

lty.ci = "solid",

graph.pos = 4,

ci.vertices = F,

txt_gp = fpTxtGp(label = gpar(fontfamily = 'Helvetica', cex = 0.9)),

xticks = c(0.7,1,1.3),

#xticks = c(-3,-2,-1,0,1,2),

graphwidth = unit(80,'mm'),

clip = c(0,6),

hrzl_lines=list,

align = c('l','c','r'))
